# Supplementary material for: The Planetary Child Health & Enterics Observatory (Plan-EO): A protocol for an interdisciplinary research initiative and web-based dashboard for mapping enteric infectious diseases and their risk factors and interventions in LMICs
Source: PLoS One. 2024 Feb 27;19(2):e0297775. doi: 10.1371/journal.pone.0297775 (PMC10898779; doi:10.1371/journal.pone.0297775)
Supplement: Attachment — Submitted filename: PONE-D-23-13586 Responses to Reviewer Comments.docx [file pone.0297775.s001.docx]

# PONE-D-23-13586 – Responses to Reviewer Comments

We thank the reviewers for their helpful reviews. We have addressed each comment below with our response (indented and in italics). As requested, we have provided both a marked-up copy of the amended manuscript as well as an unmarked version with the changes incorporated. Note that we have provided an updated version of figure 3 and, since this was created by a new member of our team, we have added that individual as an additional co-author. Responding to this review has significantly improved the manuscript and we ask you to again consider this for publication in *PLOS ONE*

## Journal Requirements:

1. Please ensure that your manuscript meets PLOS ONE's style requirements, including those for file naming.

*We have amended the formatting of our manuscript in accordance with the style requirements.*

2. We note that the grant information you provided in the ‘Funding Information’ and ‘Financial Disclosure’ sections do not match. When you resubmit, please ensure that you provide the correct grant numbers for the awards you received for your study in the ‘Funding Information’ section.

*We have amended the funding information to match the financial disclosure section.*

3. Your ethics statement should only appear in the Methods section of your manuscript. If your ethics statement is written in any section besides the Methods, please delete it from any other section.

*We have removed the ethics statement that was previously at the end of the manuscript and integrated with the “ethical considerations” section of the methods.*

4. We noted in your submission details that a portion of your manuscript may have been presented or published elsewhere. [The manuscript is available as a preprint in Research Square (<https://www.researchsquare.com/article/rs-2640564/v2>). The datasets used to generate figure 2 are all from previous publications which are cited within the figure legend.] Please clarify whether this [conference proceeding or publication] was peer-reviewed and formally published. If this work was previously peer-reviewed and published, in the cover letter please provide the reason that this work does not constitute dual publication and should be included in the current manuscript.

*The preprint in Research Square was not peer-reviewed and will be taken down upon publication of this manuscript. A poster presentation of this protocol was presented at the ASTMH 2023 Annual Meeting but has not been published in any publicly available conference proceedings. All data used in the manuscript are for illustrative purposes only, do not represent new results, and their sources are cited throughout. The exception to this is Figure 2 panel a. which shows preliminary findings from an unfinished analysis by our group (publication forthcoming). Again, this is presented for illustrative purposes and the details of the data will likely change considerably before they are formally published. Therefore, nothing in this work constitutes dual publication.*

5. In your Data Availability statement, you have not specified where the minimal data set underlying the results described in your manuscript can be found. PLOS defines a study's minimal data set as the underlying data used to reach the conclusions drawn in the manuscript and any additional data required to replicate the reported study findings in their entirety. All PLOS journals require that the minimal data set be made fully available. For more information about our data policy, please see <http://journals.plos.org/plosone/s/data-availability>. Upon re-submitting your revised manuscript, please upload your study’s minimal underlying data set as either Supporting Information files or to a stable, public repository and include the relevant URLs, DOIs, or accession numbers within your revised cover letter. For a list of acceptable repositories, please see <http://journals.plos.org/plosone/s/data-availability#loc-recommended-repositories>. Any potentially identifying patient information must be fully anonymized.

Important: If there are ethical or legal restrictions to sharing your data publicly, please explain these restrictions in detail. Please see our guidelines for more information on what we consider unacceptable restrictions to publicly sharing data: <http://journals.plos.org/plosone/s/data-availability#loc-unacceptable-data-access-restrictions>. Note that it is not acceptable for the authors to be the sole named individuals responsible for ensuring data access.

We will update your Data Availability statement to reflect the information you provide in your cover letter.

*We do not describe any new results in this manuscript and therefore there is no underlying data to make available that has not already been published elsewhere and cited in the manuscript (exception noted in response to comment 4 notwithstanding). This manuscript is a protocol describing the processes by which data will be compiled, analyzed, and curated in a repository. It does not purport to describe these data or results themselves. We have amended the data availability statement to reflect this.*

6. We note that you have stated that you will provide repository information for your data at acceptance. Should your manuscript be accepted for publication, we will hold it until you provide the relevant accession numbers or DOIs necessary to access your data. If you wish to make changes to your Data Availability statement, please describe these changes in your cover letter and we will update your Data Availability statement to reflect the information you provide.

*Again, there is no data specific to this manuscript that can be made available. There may be some confusion on this point since the protocol describes a data repository that does not yet exist. What we state is that as the Plan-EO initiative progresses, we pledge to make the data generated in subsequent analyses available through the online portal once it is set up and as they are published. We have amended the data availability statement to avoid confusion.*

7. We note that Figures 2 and 3 in your submission contain map images which may be copyrighted. All PLOS content is published under the Creative Commons Attribution License (CC BY 4.0), which means that the manuscript, images, and Supporting Information files will be freely available online, and any third party is permitted to access, download, copy, distribute, and use these materials in any way, even commercially, with proper attribution. For these reasons, we cannot publish previously copyrighted maps or satellite images created using proprietary data, such as Google software (Google Maps, Street View, and Earth). For more information, see our copyright guidelines: <http://journals.plos.org/plosone/s/licenses-and-copyright>. We require you to either (1) present written permission from the copyright holder to publish these figures specifically under the CC BY 4.0 license, or (2) remove the figures from your submission:

*The base maps in figure 2 were created by the authors using shapefiles that are made publicly available without restrictions. We have now cited the sources of those. We have replaced figure 3 with a new version that only uses OpenStreetMap data in the basemap.*

## Reviewer #1:

1. This manuscript deals with the presentation and proposition of a protocol for monitoring child health conditions related to diarrheal diseases through the interdisciplinarity of related sciences such as medicine, biology, hydrology, geology, etc. The authors present a manuscript proposing a project or describing a project, supported by recent literature, with pertinent, current, and interrelated sources to the various sciences. The language of the drafting of the project is clear, objective, and appropriate to the context of the proposition, integrating information from different scientific bases. In relation to the objectives and the methodology, the scope of the proposal is of a broad scope and transcends current contexts, addressing medium and long-term results for child health, specifically neglected diseases of diarrheal causes. As part of the methodology and presentation of results, and consequently, of the discussion of the findings and the proposition of the study, the authors present the theoretical and methodological integration in a new phase, differentiating itself from common approaches, thus characterizing the expansion of the scope and the evolution of investigations in the health aspect. The proposal is very well supported by the need for a study in the context of child health and the need for a new study in the field of health, especially transcending linear relationships based on a single scientific basis or science, but rather on the interdisciplinarity of the health context. It is, therefore, a bold, up-to-date, and extremely necessary proposal, which will certainly benefit the readers of the journal, whether planning studies in other areas with the same modeling, or participating in the Observatory, as well as the health of the children of this world.

*We thank the reviewer for their incisive and commendatory characterization of our manuscript.*

## Reviewer #2:

1. The underlying scientific logic presented is sound: that the availability of etiology-specific diarrheal diseases, occurrence data could enable better investment of scientific resources in vaccines. Rational is well argued and the authors present a strong justification for filling the gap in terms of a lack of an EID-specific dashboard, especially one that curates good quality geospatial data with a focus on etiology and other attributes relating to EIDs. This in turn could enable various actors to better understand and address these problems.

*We thank the review for this insightful commentary on the objectives and methods we propose in our manuscript.*

2. In the Methods section, the authors specify that they will prefer studies that collected large numbers of samples without specifying what such a number that they look for. It is useful to specify this number. So that while applying the criteria, they can be objective in whether they shall include a study or not.

*Thank you. We have specified 500 samples as a suitable threshold for prioritization.*

3. One of the approaches that the authors could consider is to try and have a maximum possible engagement of various academic and intersectoral stakeholders in choosing of the variable/covariates. In the steps indicated in the methodology, especially in Step A, B and C in the environmental spatial covariates and the subject and household level covariates given that selection of these covariates and variables requires disciplinary/domain/sectoral expertise in multiple disciplines/sectors using CO-production approaches wherever feasible with decision-makers/policymakers, urban/rural local government planners as well as with academia in the various relevant disciplines is worth considering. There have been one health and planetary health studies that have indeed demonstrated the use of CO-production approaches in these kinds of initiatives.

*Thank you for this important suggestion. We have added some text addressing this in the “International consortium of investigators” section.*

4. Another aspect that the study authors need to consider is to allude to potential overlaps/linkages with various emerging inter-sectoral frameworks/approaches being used in public health, such as onehealth, health in all policies, intersectoral Action for Health etc. Often, planetary health appears to be attempting to address this same challenge of working across sectors (as opposed to working in sectoral/disciplinary silos).

*We have added a sentence addressing this point in the last paragraph of the discussion section.*

5. In the discussion section, the author's mentioned an interactive dashboard, the technology platform underlying the visualization aspect of such a dashboard has not been provided. In the interest of receiving critique/generating post-publication engagement on the protocol, disclosing probable platforms on which such a dashboard is going to be posted could be helpful for the wider community that is engaging in such initiatives.

*We have now specified the probable platform (the* shiny *web application framework for R) in the methods section.*
